# Effect of prior female SARS-CoV-2 infection on IVF outcomes: a prospective cohort study

**DOI:** 10.3389/fendo.2023.1239903

**Published:** 2023-10-04

**Authors:** Jialyu Huang, Yuxin Liu, Leizhen Xia, Yan Zhao, Lifeng Tian, Dingfei Xu, Qiong Su, Yina Hu, Qiqi Xie, Jia Chen, Yunjun Li, Xiaoyan Ai, Jiawei Wang, Qiongfang Wu

**Affiliations:** ^1^ Center for Reproductive Medicine, Jiangxi Key Laboratory of Women’s Reproductive Health, Jiangxi Maternal and Child Health Hospital, Jiangxi Branch of National Clinical Research Center for Obstetrics and Gynecology, Nanchang Medical College, Nanchang, China; ^2^ Department of Clinical Medicine, School of Queen Mary, Nanchang University, Nanchang, China; ^3^ Department of Gynecology, Jiangxi Maternal and Child Health Hospital, Jiangxi Branch of National Clinical Research Center for Obstetrics and Gynecology, Nanchang Medical College, Nanchang, China; ^4^ Reproductive and Genetic Hospital, The First Affiliated Hospital of USTC, Division of Life Sciences and Medicine, University of Science and Technology of China, Hefei, China

**Keywords:** COVID-19, SARS-CoV-2, infection, pregnancy, *in vitro* fertilization

## Abstract

**Introduction:**

The clinical impact of SARS-CoV-2 infection on human reproduction remains controversial. This prospective cohort study aimed to assess the effect of prior female SARS-CoV-2 infection on subsequent *in vitro* fertilization (IVF) outcomes.

**Materials and methods:**

A total of 451 women who underwent fresh IVF treatment between December 1, 2022 and April 30, 2023 were included from an academic fertility center. Participants were divided into the infected group if they had a prior COVID-19 history before cycle initiation (n = 252), while the control group were those uninfected (n = 199). The primary outcomes were the number of oocytes retrieved and clinical pregnancy rate after fresh embryo transfer. Multivariate linear and logistic regression analyses were conducted to control for potential confounders.

**Results:**

The number of oocytes retrieved (11.4 ± 8.3 vs. 11.6 ± 7.7; P = 0.457) and clinical pregnancy rate (70.3% vs. 73.7%; P = 0.590) were similar between infected and uninfected groups, with a fully adjusted β coefficient of 0 (95% confidence interval [CI]: -0.14–0.13) and odds ratio of 0.64 (95% CI: 0.20–2.07), respectively. Consistently, the two groups were comparable in cycle characteristics as well as other laboratory and pregnancy parameters. In both subgroup analyses and restricted cubic splines, different post-infection time intervals to IVF cycle initiation showed no significant associations with treatment outcomes.

**Conclusion:**

Prior SARS-CoV-2 infection in females had no adverse influence on subsequent IVF treatment, regardless of the time interval following infection. Our findings provide reassurance for infected women planning for assisted reproduction. Additional prospective cohort studies with larger datasets and longer follow-up are required to confirm the conclusion.

## Introduction

Coronavirus disease 2019 (COVID-19) is caused by severe acute respiratory syndrome coronavirus 2 (SARS-CoV-2) (1). It was first reported in China and then become a global pandemic after a rapid spread (2). More than 600 million cumulative cases have been confirmed and over 2.5 million people have died because of this disease (3).

SARS-CoV-2 gains entry into host cells by attaching its spike (S) protein to angiotensin-converting enzyme 2 (ACE2) receptors on the cell surface (4, 5), which is then cleaved by transmembrane protease serine 2 (TMPRSS2) and being activated (4, 6). Results of single-cell RNA sequencing have demonstrated that both ovary and uterus have a co-expression of these entry factors, implying their susceptibility to infection (7–9). In the ovary, ACE2 plays a vital role in the regulation of steroidogenesis, follicle development and oocyte maturation (10, 11), and is also required for endometrial stromal decidualization (12). Additionally, *in vitro* experiments have proven a cytopathic effect of SARS-CoV-2 on human blastocyst, ranging from focal degradation to global collapse (13). These findings suggest that SARS-CoV-2 infection may negatively affect female fertility and the outcomes of assisted reproductive treatment.

To clarify the post-infection impact, an increasing number of clinical cohorts have made explorations. An earlier study showed that serum anti-Müllerian hormone (AMH) levels were significantly lower in COVID-19 women of reproductive age compared with matched controls (14). Nonetheless, several other larger studies revealed no significant correlation with ovarian reserve (15–18). Regarding the effect on *in vitro* fertilization (IVF) treatment, the first published cohort with seven women found reduced top-quality embryos after SARS-CoV-2 infection (19). Subsequently, Herrero et al. (15) observed decreased number of retrieved oocytes among infected women >35 years, while Chico-Sordo et al. (20) demonstrated that the oocyte maturation rate was lower in severe cases. On the contrary, results from other cohorts showed no significant effect (21–24). These controversial results, derived from studies with relatively small sample size, imply the need of further research for investigation.

On December 2022, the Chinese government released “a circular on further optimizing the COVID-19 response”. This shift marked the relaxation of the dynamic zero-COVID policy, and led to a widespread infection. In this context, the aim of the present study was to thoroughly assess the effect of prior SARS-CoV-2 infection on subsequent IVF cycle outcomes in a prospective cohort of Chinese infertile women.

## Materials and methods

### Study design and participants

This prospective cohort study was conducted at the Center for Reproductive Medicine, Jiangxi Maternal and Child Health Hospital affiliated to Nanchang Medical College. The study received approval from the Reproductive Medicine Ethics Committee of Jiangxi Maternal and Child Health Hospital (No. 2022-10) and adhered to the Declaration of Helsinki. All women provided written informed contents for data collection and anonymous use in scientific research.

From December 1, 2022 to April 30, 2023, all infertile women attending our center for fresh IVF treatment were approached for eligibility. Participant were divided into the infected group if they had a prior COVID-19 history before cycle initiation, while the control group were those uninfected. For the study purpose, women with SARS-CoV-2 infection during ovarian stimulation or after embryo transfer were not included. Other exclusion criteria were: 1) age ≥45 years old; 2) history of recurrent pregnancy loss or repeated implantation failure; 3) donor sperm or oocyte cycle; 4) preimplantation genetic testing cycle; and 5) lost to follow-up or missing data in the electronic health record. For women undergoing multiple IVF cycles during the timeframe, only the first cycles were retained for analysis.

The diagnosis of COVID-19 was determined by positive rapid SARS-CoV-2 antigen test or real-time RT-PCR assay in nasopharyngeal swabs, while non-infection was confirmed by negative RT-PCR test. In compliance with the tenth edition of the Diagnosis and Treatment Protocol for COVID-19 issued by the National Health Commission of China (25), we further made classifications on the clinical severity of infected women, including asymptomatic, mild, moderate, severe, and critical. The vaccination status was also collected via immunization records as previously described (26).

### IVF protocol

Controlled ovarian stimulation was performed in a depot gonadotropin-releasing hormone agonist long regimen, a flexible antagonist regimen, or other regimens such as progestin-primed stimulation. The initial gonadotropin dosage was mainly based on female age, body mass index (BMI), and markers of ovarian reserve including AMH, antral follicle count, and basal follicle-stimulating hormone (FSH) level. During stimulation, follicular development was monitored by transvaginal ultrasound and serum hormone measurement, and gonadotropin dosage was further modified. As soon as the largest follicle reached 20 mm or two dominant follicles reached a mean diameter of 18 mm, ovulation was triggered by the administration of 250 μg recombinant human chorionic gonadotropin (hCG; approval number S20130091, Ovidrel, Merck Serono, Switzerland).

After triggering, follicular aspiration was planned 36 to 38 hours later. According to the semen quality and fertilization outcome in previous cycles, conventional IVF and/or intracytoplasmic sperm injection (ICSI) was used to inseminate the retrieved oocytes. Evaluation of pronuclei (PN) was conducted 16–18 hours after insemination. The zygotes were sequentially cultivated in G1-plus medium (catalog number 10128, Vitrolife, Sweden) to day 3 cleavage-stage embryos, and then switched to G2-plus medium (catalog number 10132, Vitrolife, Sweden) for blastocyst culture until day 5 or 6. On day 3, those with ≥7 even blastomeres, ≤15% fragmentation, and no multinucleation and vacuoles were defined as good-quality embryos according to the Cummins’ morphological criteria (27). Blastocysts were graded using the Gardner and Schoolcraft system, and those with an expansion score ≥3 and inner cell mass or trophectoderm score ≥B were considered to be available (28).

After oocyte retrieval, 60 mg progesterone (approval number H33020828, Xianju Pharma, China) was injected per day to induce endometrial transformation of women undergoing fresh embryo transfer. Depending on the developmental stage, up to two embryos were transferred three or five days later via the direction of transabdominal ultrasound. Daily luteal phase support was provided in a combination of 90 mg progesterone gel vaginally (approval number H20140552, Crinone, Merck Serono, Switzerland) and 20 mg dydrogesterone orally (approval number HJ20170221, Duphaston, Abbott Biologicals, USA), and continued until 10 gestational weeks. Other embryos were vitrified for subsequent thawing and transfer.

### Outcome measures

For laboratory results, the oocyte retrieval rate was calculated by dividing the number of oocytes retrieved by the number of follicles ≥14 mm on trigger day. The ICSI mature oocyte rate and normal fertilization rate were determined by dividing the number of metaphase II (MII) and 2PN oocytes by the total number of oocytes, respectively. The cleavage rate was obtained by dividing the number of day 3 cleavage-stage embryos produced from 2PN oocytes by the total number of 2PN oocytes. The good-quality embryo rate was calculated by dividing the number of good-quality embryos on day 3 by the total number of embryos at the cleavage stage. The blastocyst formation rate was calculated by dividing the number of blastocysts by the number of day 3 embryos subjected to extended culture. Finally, the available blastocyst rate was calculated by dividing the number of available blastocysts by the total number of blastocysts on days 5 and 6.

Regarding pregnancy outcomes, biochemical pregnancy was defined as a serum β-hCG level of ≥5 mIU/mL at 10–12 days after embryo transfer. Clinical pregnancy was determined as the discovery of at least one gestational sac with or without fetal heart beat at 1 month following transfer. The number of gestational sacs divided by the number of embryos transferred was used to measure the implantation rate.

### Statistical analyses

Continuous variables were summarized as means with standard deviations and assessed for normality using the Shapiro-Wilk and Kolmogorov-Smirnov tests. Normally distributed data were compared using Student’s t-test or one-way analysis of variance (ANOVA), while skewed data were analyzed using the Mann-Whitney U-test or Kruskal-Wallis test. Categorical variables were presented as numbers and percentages, and comparisons were made using Pearson’s Chi-square test or Fisher’s exact test when appropriate.

Multivariate regression analyses were conducted to assess the independent effect of prior SARS-CoV-2 infection on IVF outcomes. Linear regression models were used to estimate β coefficients and 95% confidence intervals (CIs) for the laboratory outcomes. Adjusted variables included age, BMI, infertility duration, type and diseases, AFC, AMH, basal FSH level, previous IVF attempts, ovarian stimulation regimen, and vaccination status without or with male infection. Logistic regression models were used to calculate odds ratios (ORs) and 95% CIs for pregnancy outcomes. In addition to the aforementioned covariates, we also controlled for the number, stage, and quality of transferred embryo in the adjusted analysis.

To evaluate time-associated changes, subgroup analyses were further carried out by dividing infected women according to the interval from infection to IVF treatment: ≤30 days, 31–60 days, and >60 days. Restricted cubic splines were also used to depict the relationship between time interval as a continuous variable and the primary outcomes of retrieved oocyte number and clinical pregnancy rate.

Data analyses were performed using SAS version 9.4 (SAS Institute, USA) and R version 4.0.2 (R Foundation, USA). All tests were 2-sided and P <0.05 was considered as statistically significant.

## Results

In total, 451 eligible women were included in the analysis, including 252 (55.9%) with a prior COVID-19 history and 199 (44.1%) without. For infected women, the mean time interval to IVF treatment was 63.4 ± 21.9 days. Most were mildly to moderately infected (n = 230; 91.3%), while a minority were asymptomatic (n = 22; 8.7%). No participants were lost to follow-up or withdrew from the cohort.

**Table 1** displays the baseline and clinical characteristics according to the status of infection. Compared with the control group, the infected group had a consistently higher proportion of male infection (92.1% vs. 10.2%; P <0.001) and a slightly lower percentage of female vaccination (92.5% vs. 96.5%; P = 0.069). No significant differences were observed in age, BMI, ovarian reserve, infertility duration, type and diseases, previous IVF attempts, stimulation regimen, and fertilization method.

**Table 1:** Baseline and clinical characteristics of infected and uninfected women.

	Infected (n = 252)	Control (n = 199)	P-value
Age (years)	32.3 ± 5.0	32.2 ± 4.9	0.848
Body mass index (kg/m2)	22.2 ± 2.9	22.4 ± 3.1	0.349
Infertility duration (years)	3.7 ± 3.3	3.5 ± 3.0	0.698
Type of infertility, n (%)			0.290
Primary	94 (37.3)	84 (42.2)	
Secondary	158 (62.7)	115 (57.8)	
Infertility diseases			
Tubal factor, n (%)	124 (49.2)	100 (50.3)	0.826
Male factor, n (%)	67 (26.6)	51 (25.6)	0.818
Ovulatory dysfunction, n (%)	39 (15.5)	25 (12.6)	0.379
Diminished ovarian reserve, n (%)	79 (31.4)	52 (26.1)	0.226
Endometriosis, n (%)	19 (7.5)	11 (5.5)	0.395
Uterine factor, n (%)	25 (9.9)	28 (14.1)	0.174
Antral follicle count	12.4 ± 7.6	13.2 ± 7.8	0.308
AMH (ng/mL)	3.1 ± 3.1	3.2 ± 3.2	0.477
Basal FSH level (mIU/mL)	7.3 ± 5.5	6.9 ± 2.9	0.748
Previous IVF attempts, n (%)			0.087
0	209 (82.9)	164 (82.4)	
1-2	29 (11.5)	31 (15.6)	
≥3	14 (5.6)	4 (2.0)	
Ovarian stimulation regimen, n (%)			0.516
GnRH agonist	155 (61.5)	129 (64.8)	
GnRH antagonist	50 (19.8)	41 (20.6)	
Others	47 (18.7)	29 (14.6)	
Fertilization method, n (%)			0.648
IVF	167 (66.3)	134 (67.3)	
ICSI	75 (29.8)	54 (27.1)	
IVF+ICSI	10 (4.0)	11 (5.5)	
Vaccinated, n (%)	233 (92.5)	192 (96.5)	0.069
Male infection, n (%)	232 (92.1)	22 (10.2)	<0.001

Data are presented as mean ± standard deviation or number (percentage).

AMH, anti-Müllerian hormone; FSH, follicle-stimulating hormone; GnRH, gonadotropin-releasing hormone; IVF, in vitro fertilization; ICSI, intracytoplasmic sperm injection.

Cycle characteristics and laboratory outcomes grouped by the infection status are demonstrated in **Table 2**. There were no significant differences regarding the stimulation duration, total gonadotropin dose, serum estradiol level, endometrial thickness, and number of ≥14 mm follicles on trigger day. The number of oocytes retrieved (11.4 ± 8.3 vs. 11.6 ± 7.7; P = 0.457), as well as MII oocytes, 2PN oocytes, cleaved embryos, and good-quality day 3 embryos, were all comparable between infected and uninfected women. Consistently, similar outcomes were observed in the oocyte retrieval rate, mature oocyte rate, normal fertilization rate, cleavage rate, good-quality embryo rate, blastocyst formation rate, and available blastocyst rate.

**Table 2:** Cycle characteristics and laboratory outcomes.

	Infected (n = 252)	Control (n = 199)	P-value
Stimulation duration (days)	10.3 ± 2.5	10.4 ± 2.3	0.487
Total gonadotropin dose (IU)	1874.6 ± 714.8	1918.7 ± 651.8	0.348
Estradiol level on trigger day (pg/mL)	1788.9 ± 1322.3	1791.2 ± 1347.7	0.835
Endometrial thickness on trigger day (mm)	10.4 ± 3.0	10.6 ± 2.9	0.322
No. of ≥14 mm follicles on trigger day	8.2 ± 5.2	8.4 ± 4.6	0.448
No. of oocytes retrieved	11.4 ± 8.3	11.6 ± 7.7	0.457
No. of MII oocytes (ICSI)	9.0 ± 6.1	8.7 ± 4.8	0.854
No. of 2PN oocytes	6.6 ± 5.2	6.6 ± 4.6	0.411
No. of cleaved embryos	6.3 ± 5.2	6.4 ± 4.5	0.382
No. of good-quality embryos on day 3	1.8 ± 2.3	1.6 ± 1.6	0.763
Oocyte retrieval rate (%)	138.9 ± 50.1	136 ± 52.3	0.693
ICSI mature oocyte rate (%)	72.1 ± 17.8	72.4 ± 15.9	0.586
Normal fertilization rate (%)	66.3 ± 24.4	64.8 ± 24.2	0.463
Cleavage rate (%)	96.9 ± 8.0	96.7 ± 10.3	0.946
Good-quality embryo rate (%)	28.6 ± 26.5	27.6 ± 25.8	0.681
Blastocyst formation rate (%)	72.6 ± 31.3	72.2 ± 27.0	0.322
Available blastocyst rate (%)	72.9 ± 29.0	73.6 ± 29.0	0.836

Data are presented as mean ± standard deviation.

ICSI, intracytoplasmic sperm injection; MII, metaphase II; 2PN, two pronuclei.

**Table 3** shows pregnancy outcomes after fresh embryo transfer. A total of 213 women were enrolled, comprising 118 in the infected group and 95 in the uninfected group. The number, stage, and quality of embryos transferred were comparable between groups. Clinical pregnancy rate per cycle was 70.3% and 73.7% in infected and uninfected women, respectively (P = 0.590). Likewise, the two groups did not differ significantly in biochemical pregnancy rate (74.6% vs. 82.1%; P = 0.188) and implantation rate (57.23% vs. 60.29%; P = 0.591).

**Table 3:** Pregnancy outcomes after fresh embryo transfer.

	Infected (n = 118)	Control (n = 95)	P-value
Number of embryos transferred, n (%)			0.715
1	70 (59.3)	54 (56.8)	
2	48 (40.7)	41 (43.2)	
Stage of embryos transferred, n (%)			0.798
Cleavage	48 (40.7)	37 (39)	
Blastocyst	70 (59.3)	58 (61.1)	
Transfer of good-quality embryos, n (%)	71 (60.2)	55 (57.9)	0.737
Biochemical pregnancy rate, n (%)	88 (74.6)	78 (82.1)	0.188
Clinical pregnancy rate, n (%)	83 (70.3)	70 (73.7)	0.590
Implantation rate, n/N (%)	95/166 (57.2)	82/136 (60.3)	0.591

Data are presented as number (percentage).

**Table 4** presents the association of female SARS-CoV-2 infection with laboratory and pregnancy outcomes on crude and adjusted analyses. After controlling for potential confounders excluding male infection, prior female infection had no significant impact on the number of oocytes retrieved (adjusted β = 0.06, 95% CI: -0.03–0.14) and clinical pregnancy rate (adjusted OR = 0.88, 95% CI: 0.44–1.73). When male infection was further included in the multivariate regression model, the fully adjusted β coefficient was 0 (95% CI: -0.14–0.13) and OR was 0.64 (95% CI: 0.20–2.07), respectively. Similarly, the outcomes of mature oocyte rate, normal fertilization rate, cleavage rate, good quality embryo rate, blastocyst formation rate, available blastocyst rate, and biochemical pregnancy rate remained unrelated to the infection status. As demonstrated in **Supplementary Tables S1, S2**, other influencing factors of retrieved oocyte number included age, AMH, basal FSH, diagnosis of diminished ovarian reserve and ovarian stimulation regimen, while increased age and ovulatory dysfunction were significant risk factors for lower clinical pregnancy odds.

**Table 4:** Crude and adjusted analyses on the association between prior infection and IVF outcomes.

	Crude analysis		Adjusted analysis		Adjusted analysis *	
	β/OR (95% CI)	P-value	β/OR (95% CI)	P-value	β/OR (95% CI)	P-value
Laboratory outcomes						
No. of oocytes retrieved	-0.02 (-0.15–0.11)	0.740	0.06 (-0.03–0.14)	0.174	0 (-0.14–0.13)	0.957
ICSI mature oocyte rate (%)	0 (-0.09–0.08)	0.930	0.02 (-0.07–0.10)	0.711	0 (-0.13–0.13)	0.953
Normal fertilization rate (%)	0.02 (-0.05–0.09)	0.514	0.01 (-0.05–0.08)	0.689	0.04 (-0.07–0.16)	0.458
Cleavage rate (%)	0 (-0.02–0.02)	0.825	0 (-0.02–0.02)	0.876	-0.01 (-0.04–0.02)	0.384
Good-quality embryo rate (%)	0.04 (-0.14–0.21)	0.691	0.02 (-0.16–0.19)	0.847	-0.06 (-0.34–0.22)	0.678
Blastocyst formation rate (%)	0.01 (-0.08–0.10)	0.884	0.03 (-0.06–0.12)	0.506	0.05 (-0.11–0.21)	0.539
Available blastocyst rate (%)	-0.01 (-0.1–0.08)	0.825	0 (-0.09–0.09)	0.995	-0.05 (-0.21–0.11)	0.546
Pregnancy outcomes						
Biochemical pregnancy	0.64 (0.33–1.25)	0.190	0.59 (0.27–1.31)	0.197	0.29 (0.07–1.20)	0.087
Clinical pregnancy	0.85 (0.46–1.55)	0.590	0.88 (0.44–1.73)	0.703	0.64 (0.20–2.07)	0.453

*Male infection was adjusted in addition to other covariates.

CI, confidence interval; ICSI, intracytoplasmic sperm injection; OR, odds ratio.

**Figure 1:**
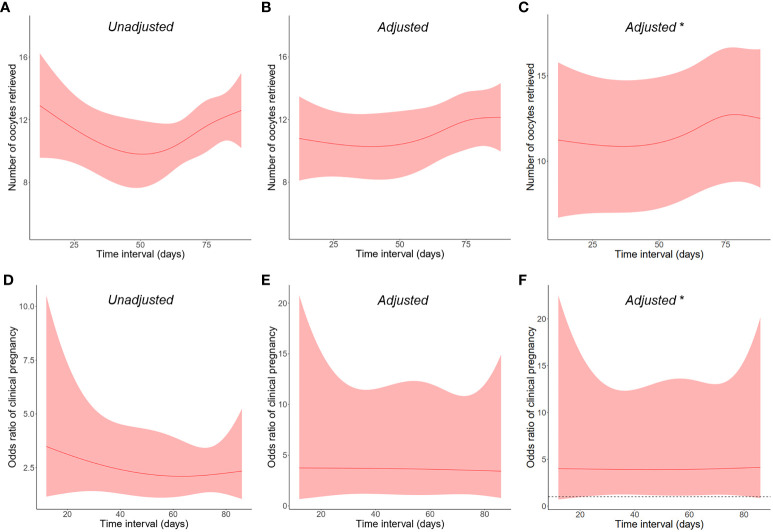
Restricted cubic splines on the relationship between post-infection time interval as a continuous variable and the primary outcomes of **(A–C)** retrieved oocyte number and **(D–F)** clinical pregnancy rate. The shaded areas represent the 95% confidence intervals. *Male infection was adjusted in addition to other covariates.

According to the time interval between SARS-CoV-2 infection and IVF cycle initiation, infected women were subdivided into three categories: ≤30 days, 31–60 days, and >60 days. As observed in **Table 5**, there were no discernible differences in all laboratory and pregnancy outcomes, including retrieved oocyte number (12.1 ± 5.8, 10.2 ± 8.1, and 11.6 ± 8.7, respectively, and 11.6 ± 7.7 in the uninfected group; P = 0.316) and clinical pregnancy rate (73.9%, 66.7%, and 70.6%, respectively, and 73.7% in the uninfected group; P = 0.892). To further clarify the pattern, the time interval was analyzed as a continuous variable, and these two primary outcomes remained similar in both crude and adjusted analyses (**Figure 1**). We also conducted a stratified analysis according to the severity of prior COVID-19, and found no significant differences between women with asymptomatic and mild-to-moderate infection (**Supplementary Table S3**).

**Table 5:** Subgroup analyses according to the time interval between SARS-CoV-2 infection and IVF treatment.

	≤30 d	31–60 d	>60 d	Control	P-value
Laboratory outcomes	n = 32	n = 54	n = 166	n = 199	
No. of oocytes retrieved	12.1 ± 5.8	10.2 ± 8.1	11.6 ± 8.7	11.6 ± 7.7	0.316
Crude β (95% CI)	0.04 (-0.20–0.29)	-0.13 (-0.36–0.10)	0 (-0.15–0.14)	1	
Adjusted β (95% CI)	-0.05 (-0.21–0.12)	0 (-0.15–0.14)	0.10 (0.01–0.18)	1	
Adjusted β (95% CI) *	-0.12 (-0.32–0.09)	-0.06 (-0.23–0.11)	0.03 (-0.11–0.17)	1	
ICSI mature oocyte rate (%)	75.4 ± 19.0	75 ± 16.9	71 ± 18.0	72.4 ± 15.9	0.707
Crude β (95% CI)	0.04 (-0.11–0.19)	0.03 (-0.11–0.18)	-0.02 (-0.11–0.07)	1	
Adjusted β (95% CI)	0.04 (-0.11–0.19)	0.03 (-0.13–0.20)	0.01 (-0.09–0.10)	1	
Adjusted β (95% CI) *	0.01 (-0.17–0.19)	0.01 (-0.17–0.20)	-0.01 (-0.15–0.12)	1	
Normal fertilization rate (%)	69.9 ± 23.1	70.8 ± 20.7	64.2 ± 25.6	64.8 ± 24.2	0.255
Crude β (95% CI)	0.08 (-0.05–0.21)	0.09 (-0.02–0.20)	-0.01 (-0.09–0.07)	1	
Adjusted β (95% CI)	0.05 (-0.07–0.18)	0.06 (-0.04–0.17)	-0.01 (-0.09–0.06)	1	
Adjusted β (95% CI) *	0.09 (-0.08–0.25)	0.09 (-0.05–0.22)	0.02 (-0.10–0.13)	1	
Cleavage rate (%)	96.6 ± 8.3	97.7 ± 7.2	96.7 ± 8.3	96.7 ± 10.3	0.631
Crude β (95% CI)	0 (-0.04–0.03)	0.01 (-0.02–0.04)	0 (-0.02–0.02)	1	
Adjusted β (95% CI)	0 (-0.03–0.04)	0.01 (-0.02–0.04)	-0.01 (-0.03–0.01)	1	
Adjusted β (95% CI) *	-0.01 (-0.05–0.03)	0 (-0.04–0.03)	-0.02 (-0.05–0.01)	1	
Good-quality embryo rate (%)	29.5 ± 24.3	32.2 ± 27.4	27.3 ± 26.6	27.6 ± 25.8	0.563
Crude β (95% CI)	0.07 (-0.27–0.40)	0.15 (-0.11–0.41)	-0.01 (-0.21–0.19)	1	
Adjusted β (95% CI)	0.14 (-0.19–0.47)	0.15 (-0.12–0.41)	-0.05 (-0.25–0.15)	1	
Adjusted β (95% CI) *	0.05 (-0.35–0.45)	0.07 (-0.25–0.39)	-0.14 (-0.43–0.15)	1	
Blastocyst formation rate (%)	69.1 ± 33.1	80.6 ± 26.6	70.6 ± 32.2	72.2 ± 27.0	0.191
Crude β (95% CI)	-0.04 (-0.21–0.13)	0.11 (-0.02–0.24)	-0.02 (-0.12–0.08)	1	
Adjusted β (95% CI)	-0.04 (-0.20–0.13)	0.11 (-0.02–0.24)	0.01 (-0.09–0.11)	1	
Adjusted β (95% CI) *	-0.03 (-0.25–0.19)	0.12 (-0.06–0.29)	0.02 (-0.15–0.18)	1	
Available blastocyst rate (%)	68.3 ± 29.4	72.3 ± 31.0	74.2 ± 28.3	73.6 ± 29	0.665
Crude β (95% CI)	-0.08 (-0.25–0.10)	-0.02 (-0.16–0.12)	0.01 (-0.09–0.11)	1	
Adjusted β (95% CI)	-0.08 (-0.25–0.10)	-0.01 (-0.15–0.13)	0.02 (-0.07–0.12)	1	
Adjusted β (95% CI) *	-0.14 (-0.37–0.09)	-0.06 (-0.25–0.12)	-0.03 (-0.19–0.13)	1	
Pregnancy outcomes	n = 23	n = 27	n = 68	n = 95	
Biochemical pregnancy rate, n (%)	17 (73.9)	18 (66.7)	53 (77.9)	78 (82.1)	0.366
Crude OR (95% CI)	0.62 (0.21–1.80)	0.44 (0.17–1.14)	0.77 (0.35–1.68)	1	
Adjusted OR (95% CI)	0.57 (0.16–2.10)	0.38 (0.12–1.24)	0.76 (0.29–1.96)	1	
Adjusted OR (95% CI) *	0.25 (0.04–1.71)	0.2 (0.04–1.03)	0.37 (0.08–1.73)	1	
Clinical pregnancy rate, n (%)	17 (73.9)	18 (66.7)	48 (70.6)	70 (73.7)	0.892
Crude OR (95% CI)	1.01 (0.36–2.85)	0.71 (0.28–1.8)	0.86 (0.43–1.72)	1	
Adjusted OR (95% CI)	1.04 (0.31–3.49)	0.94 (0.32–2.74)	0.80 (0.36–1.79)	1	
Adjusted OR (95% CI) *	0.72 (0.14–3.74)	0.71 (0.18–2.81)	0.58 (0.17–2.06)	1	
Implantation rate, n/N (%)	19/33 (57.6)	21/37 (56.8)	55/96 (57.3)	82/136 (60.3)	

*Male infection was adjusted in addition to other covariates.

CI, confidence interval; ICSI, intracytoplasmic sperm injection; OR, odds ratio.

## Discussion

The results of our prospective cohort study demonstrated that prior SARS-CoV-2 infection in females had no adverse influence on IVF treatment. Moreover, different time intervals from infection to cycle initiation did not significantly affect either laboratory or pregnancy outcomes.

Since ACE2 and TMPRSS2 receptors are expressed in the ovary, it has been speculated that SARS-CoV-2 may be harmful to female reproduction (7–9, 29–31). However, most clinical studies showed that viral RNA was undetectable in the follicular fluid (FF) and granulosa cells (32, 33). Consistently, a study with 16 mature oocytes from two SARS-CoV-2-positive women detected no viral RNA for gene N in oocytes (34). Nonetheless, SARS-CoV-2 may still alter follicular microenvironment in the absence of direct infection. For example, Herrero et al. found that the FF from post COVID-19 women had a high positivity for SARS-CoV-2 IgG antibodies and reduced VEGF and IL-1β levels. In addition, post COVID-19 FF decreased granulosa cell steroidogenesis, impaired endothelial cell migration, and severely damaged DNA stability and integrity in both cells (15). In metabolomic and lipidomic analyses, significant FF alterations were also observed among infected women (35, 36), which may lead to worse ovarian response, oocyte quality, and embryo development during IVF treatment.

Emerging cohort studies have made investigations on the clinical effect of these changes. Based on small sample sizes 7 to 80, some earlier studies found lower oocyte number, fewer top-quality embryos and decreased oocyte maturation rate in women after SARS-CoV-2 infection (15, 19, 37). The largest cohort to date, with 4043 pre-matching cycles and 260 post-matching cycles, also observed a slight reduction in blastocyst formation rate in the case group. However, no significant differences were detected in any other female fertility parameters or embryo laboratory outcomes (38). Similarly, several subsequent studies also demonstrated that there was no negative effect of prior COVID-19 history on IVF cycle outcomes (21, 24, 39, 40), which are consistent with the results of our study.

Endometrium is essential for embryo implantation and was thought to be safe from SARS-CoV-2 entry due to the low levels of ACE2 and TMPRSS2 (41). However, the endometrial physiology may be affected without direct viral invasion, as evidenced by changes in menstrual volume and cycle length after COVID-19 (42). One research found that five genes crucial for endometrial receptivity were affected by COVID-19, including COBL, GPX3, SOCS3, DOCK2 and SLC2A3 (43). Similarly, another transcriptomic study observed 163 up- and 72 down-regulated genes in endometrium of infected women, which were functionally enriched in cytokine inflammation and immune responses to viruses (43). Given the potential influence, several studies have followed up pregnancy outcomes after embryo transfer. In a prospective Russian cohort, women who experienced moderate COVID-19 were found to have a higher early miscarriage rate than uninfected women (12.0% vs. 2.9%, P = 0.002) (44). However, this finding was contradicted by other studies with no adverse effect (21, 24, 38, 40). Similarly, on the basis of 213 fresh embryo transfer cycles, the present study showed no significant differences in biochemical pregnancy rate, clinical pregnancy rate, and implantation rate, suggesting the lack of clinical effect from biological changes.

The ideal time period between infection and the start of fresh IVF treatment was discussed by limited studies, and no agreement has been established yet. A small self-controlled study with seven women showed a significant decrease in the proportion of high-quality embryos after SARS-CoV-2 infection between 8 and 92 days. Therefore, the authors recommended delaying IVF for at least 3 months to allow for the completion of prior folliculogenesis cycle (19). Contrarily, Youngster et al. (40) observed decreased oocyte yield in women with a past infection >180 days, implying a possible long-term negative effect. To add more confusion, in the study by Dolgushina et al. (44), a significantly higher proportion of poor-quality blastocysts was detected in women with an interval ≤180 days than those >180 days. In the present study, both laboratory and pregnancy outcomes were comparable among infected women with an interval of 30 days or less, 31 to 60 days, and 61 days or more. The results were also consistent between crude and adjusted analyses when time interval was visualized in restricted cubic splines as a continuous variable. Based on our finding, infected women may be assured to proceed IVF cycle at their earliest convenience, but larger cohort studies are required for confirmation.

There are some limitations that should be acknowledged. Firstly, the observational design is inherently prone to potential selection bias and confounding variables. For example, we did not classify the variants of SARS-CoV-2 (e.g., Delta and Omicron), whose evolving transmissibility and pathogenicity may have different effect on IVF outcomes. Other co-morbidities like diabetes and hypertension were also missed and should be taken into consideration in the future. Secondly, although this study is one of the largest prospective cohorts thus far, the sample size may still be inadequate especially in subgroup analysis. Moreover, the single-center setting may limit the generalizability of our finding. Thirdly, only asymptomatic and mildly to moderately infected women were included, and therefore, the conclusion should be cautiously interpreted for severe COVID-19 cases. We were also unable to further distinguish between mild and moderate infections which rely on computed tomographic scan of the chest (25). Finally, due to time constraints, the study did not complete live birth follow-up of the entire cohort. In addition, we only analyzed the pregnancy outcomes of fresh embryo transfer, while the effect on frozen-thawed embryo transfer warrants further investigation.

## Conclusion

To summarize, the current study demonstrated that prior female SARS-CoV-2 infection did not pose measurable adverse effects on subsequent IVF treatment. Moreover, both laboratory and pregnancy outcomes were comparable across different post-infection time intervals. Our finding should provide reassuring information for COVID-19 women of reproductive age. However, larger multicenter prospective cohorts are warranted to validate our conclusion and assess live birth outcome as well as offspring health.

## Data availability statement

The raw data supporting the conclusions of this article will be made available by the authors, without undue reservation.

## Ethics statement

The studies involving humans were approved by Reproductive Medicine Ethics Committee of Jiangxi Maternal and Child Health Hospital (No 2022-10). The studies were conducted in accordance with the local legislation and institutional requirements. All women provided written informed contents for data collection and anonymous use in scientific research.

## Author contributions

JH, JW and QW contributed to the study conception and design. LX, YZ, LT, DX, QS, YH, QX, JC, YJL and XA were responsible for data collection and curation. JH, YXL and LX conducted the statistical analyses. JH and YXL drafted the manuscript. XA, JW and QW supervised the project administration. All authors contributed to the article and approved the submitted version.
