# Supplementary material for: Effect of prior female SARS-CoV-2 infection on IVF outcomes: a prospective cohort study
Source: Front Endocrinol (Lausanne). 2023 Oct 4;14:1239903. doi: 10.3389/fendo.2023.1239903 (PMC10582695; doi:10.3389/fendo.2023.1239903)
Supplement: Supplementary file 1 [file Table_1.docx]

**Table S1.** Multivariable regression analysis for the number of oocytes retrieved.

|  | Wald | β (95% CI) | P-value |
| --- | --- | --- | --- |
| Age (years) | 11.10 | -0.02 (-0.03–-0.01) | 0.001 |
| Body mass index (kg/m^2^) | 0.03 | 0 (-0.02–0.01) | 0.858 |
| Infertility duration (years) | 1.68 | 0.01 (-0.01–0.03) | 0.195 |
| Type of infertility |  |  |  |
| Primary | Reference | |  |
| Secondary | 0.24 | 0.02 (-0.07–0.11) | 0.626 |
| Infertility diseases |  |  |  |
| Tubal factor (yes vs. no) | 0.39 | -0.03 (-0.12–0.06) | 0.535 |
| Male factor (yes vs. no) | 2.38 | 0.08 (-0.02–0.18) | 0.123 |
| Ovulatory dysfunction (yes vs. no) | 1.19 | -0.07 (-0.18–0.05) | 0.275 |
| Diminished ovarian reserve (yes vs. no) | 22.73 | 0.56 (0.33–0.79) | <0.001 |
| Endometriosis (yes vs. no) | 2.08 | 0.16 (-0.06–0.37) | 0.149 |
| Uterine factor (yes vs. no) | 1.46 | 0.09 (-0.06–0.23) | 0.228 |
| Antral follicle count | 0.56 | 0 (0–0.01) | 0.455 |
| AMH (ng/mL) | 32.51 | 0.04 (0.03–0.06) | <0.001 |
| Basal FSH level (mIU/mL) | 4.13 | -0.02 (-0.04–0) | 0.042 |
| Previous IVF attempts |  |  |  |
| 0 | Reference | |  |
| 1-2 | 3.44 | -0.22 (-0.46–0.01) | 0.064 |
| ≥3 | 3.79 | -0.25 (-0.49–0) | 0.052 |
| Ovarian stimulation regimen |  |  |  |
| GnRH agonist | Reference | |  |
| GnRH antagonist | 16.16 | 0.97 (0.49–1.44) | <0.001 |
| Others | 17.12 | 0.99 (0.52–1.46) | <0.001 |
| Vaccinated (yes vs. no) | 1.20 | 0.10 (-0.08–0.28) | 0.273 |
| Male infection (yes vs. no) | 1.26 | -0.08 (-0.21–0.06) | 0.262 |
| Female infection (yes vs. no) | 0 | 0 (-0.14–0.13) | 0.957 |

**Abbreviations:** AMH, anti-Müllerian hormone; CI, confidence interval; FSH, follicle-stimulating hormone; GnRH, gonadotropin-releasing hormone; IVF, in vitro fertilization.

**Table S2.** Multivariable regression analysis for clinical pregnancy after fresh embryo transfer.

|  | Wald | OR (95% CI) | P-value |
| --- | --- | --- | --- |
| Age (years) | 6.63 | 1.12 (1.03–1.23) | 0.010 |
| Body mass index (kg/m^2^) | 0.12 | 1.02 (0.90–1.16) | 0.727 |
| Infertility duration (years) | 2.66 | 0.87 (0.74–1.03) | 0.103 |
| Type of infertility |  |  |  |
| Primary | Reference |  |  |
| Secondary | 0.43 | 0.76 (0.34–1.72) | 0.513 |
| Infertility diseases |  |  |  |
| Tubal factor (yes vs. no) | 0.61 | 0.74 (0.35–1.57) | 0.436 |
| Male factor (yes vs. no) | 1.80 | 0.55 (0.23–1.31) | 0.180 |
| Ovulatory dysfunction (yes vs. no) | 4.87 | 0.19 (0.04–0.83) | 0.027 |
| Diminished ovarian reserve (yes vs. no) | 1.81 | 2.46 (0.66–9.12) | 0.178 |
| Endometriosis (yes vs. no) | 3.48 | 3.27 (0.94–11.36) | 0.062 |
| Uterine factor (yes vs. no) | 2.97 | 0.36 (0.11–1.15) | 0.085 |
| Antral follicle count | 0.12 | 1.02 (0.92–1.12) | 0.726 |
| AMH (ng/mL) | 0.75 | 1.09 (0.90–1.31) | 0.387 |
| Basal FSH level (mIU/mL) | 2.07 | 1.13 (0.96–1.32) | 0.151 |
| Previous IVF attempts |  |  |  |
| 0 | Reference |  |  |
| 1-2 | 0.00 | 0.99 (0.31–3.15) | 0.979 |
| ≥3 | 0.50 | 0.26 (0.01–10.85) | 0.482 |
| Ovarian stimulation regimen |  |  |  |
| GnRH agonist | Reference |  |  |
| GnRH antagonist | 0.53 | 2.39 (0.23–24.92) | 0.468 |
| Vaccinated (yes vs. no) | 0.02 | 0.90 (0.19–4.28) | 0.899 |
| Male infection (yes vs. no) | 0.43 | 0.67 (0.21–2.21) | 0.513 |
| Female infection (yes vs. no) | 0.56 | 0.64 (0.20–2.07) | 0.453 |
| Number of embryos transferred |  |  |  |
| 1 | Reference |  |  |
| 2 | 0.24 | 0.71 (0.18–2.76) | 0.623 |
| Stage of embryos transferred |  |  |  |
| Cleavage | Reference |  |  |
| Blastocyst | 1.74 | 0.40 (0.10–1.56) | 0.188 |
| Transfer of good-quality embryos (yes vs. no) | 0.01 | 0.96 (0.46–2.00) | 0.907 |

**Abbreviations:** AMH, anti-Müllerian hormone; OR, odds ratio; CI, confidence interval; FSH, follicle-stimulating hormone; GnRH, gonadotropin-releasing hormone; IVF, in vitro fertilization.

**Table S3.** Subgroup analyses according to the severity of infection.

|  | Asymptomatic | Mild-to-moderate | Control | P-value |
| --- | --- | --- | --- | --- |
| **Laboratory outcomes** | **n = 22** | **n = 230** | **n = 199** |  |
| No. of oocytes retrieved | 12.7±9.8 | 11.2±8.1 | 11.6±7.7 | 0.612 |
| Crude β (95% CI) | 0.09 (-0.19–0.37) | -0.03 (-0.17–0.10) | 1 |  |
| Adjusted β (95% CI) | 0.12 (-0.05–0.29) | 0.05 (-0.04–0.13) | 1 |  |
| Adjusted β (95% CI) * | 0.06 (-0.14–0.26) | -0.01 (-0.15–0.12) | 1 |  |
| ICSI mature oocyte rate (%) | 80.4±22.4 | 71.4±17.4 | 72.4±15.9 | 0.526 |
| Crude β (95% CI) | 0.10 (-0.07–0.28) | -0.01 (-0.10–0.07) | 1 |  |
| Adjusted β (95% CI) | 0.06 (-0.15–0.26) | 0.01 (-0.07–0.10) | 1 |  |
| Adjusted β (95% CI) * | 0.04 (-0.20–0.27) | 0 (-0.13–0.13) | 1 |  |
| Normal fertilization rate (%) | 66.4±21.1 | 66.3±24.8 | 64.8±24.2 | 0.713 |
| Crude β (95% CI) | 0.02 (-0.14–0.19) | 0.02 (-0.05–0.09) | 1 |  |
| Adjusted β (95% CI) | -0.01 (-0.16–0.15) | 0.02 (-0.05–0.08) | 1 |  |
| Adjusted β (95% CI) * | 0.02 (-0.16–0.2) | 0.04 (-0.07–0.16) | 1 |  |
| Cleavage rate (%) | 94.5±10.6 | 97.1±7.7 | 96.7±10.3 | 0.434 |
| Crude β (95% CI) | -0.02 (-0.07–0.02) | 0 (-0.01–0.02) | 1 |  |
| Adjusted β (95% CI) | -0.03 (-0.07–0.01) | 0 (-0.02–0.02) | 1 |  |
| Adjusted β (95% CI) * | -0.05 (-0.09–0) | -0.01 (-0.04–0.02) | 1 |  |
| Good-quality embryo rate (%) | 21.2±23.0 | 29.4±26.7 | 27.6±25.8 | 0.307 |
| Crude β (95% CI) | -0.27 (-0.80–0.27) | 0.06 (-0.12–0.24) | 1 |  |
| Adjusted β (95% CI) | -0.32 (-0.89–0.25) | 0.04 (-0.13–0.21) | 1 |  |
| Adjusted β (95% CI) * | -0.4 (-1.01–0.21) | -0.04 (-0.32–0.24) | 1 |  |
| Blastocyst formation rate (%) | 74.8±23.8 | 72.4±31.9 | 72.2±27.0 | 0.590 |
| Crude β (95% CI) | 0.04 (-0.17–0.25) | 0 (-0.09–0.10) | 1 |  |
| Adjusted β (95% CI) | 0.06 (-0.14–0.27) | 0.03 (-0.06–0.12) | 1 |  |
| Adjusted β (95% CI) * | 0.08 (-0.16–0.32) | 0.05 (-0.12–0.21) | 1 |  |
| Available blastocyst rate (%) | 65.6±29.9 | 73.6±28.9 | 73.6±29.0 | 0.508 |
| Crude β (95% CI) | -0.11 (-0.35–0.12) | 0 (-0.09–0.09) | 1 |  |
| Adjusted β (95% CI) | -0.06 (-0.28–0.17) | 0.01 (-0.08–0.10) | 1 |  |
| Adjusted β (95% CI) * | -0.1 (-0.36–0.15) | -0.04 (-0.2–0.12) | 1 |  |
| **Pregnancy outcomes** | **n = 10** | **n = 108** | **n = 95** |  |
| Biochemical pregnancy rate, n (%) | 7 (70.0) | 81 (75.0) | 78 (82.1) | 0.393 |
| Crude OR (95% CI) | 0.51 (0.12–2.17) | 0.65 (0.33–1.29) | 1 |  |
| Adjusted OR (95% CI) | 0.37 (0.07–2.14) | 0.62 (0.28–1.41) | 1 |  |
| Adjusted OR (95% CI) * | 0.22 (0.03–1.73) | 0.31 (0.07–1.35) | 1 |  |
| Clinical pregnancy rate, n (%) | 7 (70.0) | 76 (70.4) | 70 (73.7) | 0.864 |
| Crude OR (95% CI) | 0.83 (0.20–3.47) | 0.85 (0.46–1.57) | 1 |  |
| Adjusted OR (95% CI) | 0.77 (0.16–3.79) | 0.89 (0.44–1.79) | 1 |  |
| Adjusted OR (95% CI) * | 0.61 (0.10–3.59) | 0.64 (0.19–2.17) | 1 |  |
| Implantation rate, n/N (%) | 8/13 (61.5） | 87/153 (56.9） | 82/136 (60.3） | 0.820 |

* Male infection was adjusted in addition to other covariates.

**Abbreviations:** CI, confidence interval; ICSI, intracytoplasmic sperm injection; OR, odds ratio.
